# Networked Roadside Perception Units for Autonomous Driving

**DOI:** 10.3390/s20185320

**Published:** 2020-09-17

**Authors:** Manabu Tsukada, Takaharu Oi, Masahiro Kitazawa, Hiroshi Esaki

**Affiliations:** Graduate School of Information Science and Technology, The University of Tokyo, 7-3-1 Hongo, Bunkyo-ku, Tokyo 113-8656, Japan; oi@hongo.wide.ad.jp (T.O.); ktzw@hongo.wide.ad.jp (M.K.); hiroshi@wide.ad.jp (H.E.)

**Keywords:** cooperative ITS, autonomous vehicle, cooperative automated vehicles (CAV), V2X, cooperative perception, open-source software

## Abstract

Vehicle-to-Everything (V2X) communication enhances the capability of autonomous driving through better safety, efficiency, and comfort. In particular, sensor data sharing, known as cooperative perception, is a crucial technique to accommodate vulnerable road users in a cooperative intelligent transport system (ITS). In this paper, we describe a roadside perception unit (RSPU) that combines sensors and roadside units (RSUs) for infrastructure-based cooperative perception. We propose a software called AutoC2X that we designed to realize cooperative perception for RSPUs and vehicles. We also propose the concept of networked RSPUs, which is the inter-connection of RSPUs along a road over a wired network, and helps realize broader cooperative perception. We evaluated the RSPU system and the networked RSPUs through a field test, numerical analysis, and simulation experiments. Field evaluation showed that, even in the worst case, our RSPU system can deliver messages to an autonomous vehicle within 100 ms. The simulation result shows that the proposed priority algorithm achieves a wide perception range with a high delivery ratio and low latency, especially under heavy road traffic conditions.

## 1. Introduction

Road transport infrastructure is an essential part of modern human life. However, it currently poses many issues, such as accidents, high energy consumption, time loss, and CO_2_ emission. Intelligent Transportation System (ITS) objectives include road traffic optimization in terms of safety, efficiency, and comfort. In recent years, among the many technologies associate with the domain of ITS, autonomous vehicles have attracted the most attention of researchers. Autonomous vehicles use a set of sensors such as LiDAR systems, cameras, and global navigation system satellite (GNSS)/inertial measurement units to perceive the surrounding environment. Using the sensor data, the autonomous driving system accelerates, steers, and brakes without the help of human input. However, at a more abstract level, standalone autonomous vehicles are not significantly different from conventional vehicles in that they merely replace human eyes, brain, arms, and legs with sensors, processors, and actuators. Hence, both human drivers and autonomous vehicles share some limitations in terms of their capability of perception, planning, and control. Especially with regard to perception capability, both eyes and sensors have very similar fields of view because they are both positioned on the vehicle, and heavy vehicles and buildings affect both in the same way by obstructing their line of sight (LOS). These shared limitations prevent autonomous vehicles from a safer or more efficient driving operation than human drivers. For example, both need to stop in front of an unsignalized intersection and approach it slowly. In theory, it would be safer and more efficient to move in the intersection without stopping by shortening the time in the intersection.

Vehicle-to-everything (V2X) communication connects vehicles, roadside infrastructure, pedestrians, and networks, and has the potential to overcome some of the issues described above through better perception of the surrounding environment. Two types of wireless technologies have been under research and development for V2X: Dedicated short-range communication (DSRC) and cellular V2X. Cooperative ITS refers to transport systems, where the cooperation between two or more ITS sub-systems using these V2X technologies. Therefore, cooperative ITS needs the same architecture, technologies, and protocols for interoperability among the products and software in the market. The common architecture, known as the ITS station architecture [1,2], was developed by European Telecommunications Standards Institute (ETSI) and International Organization for Standardization (ISO). According to this standard, a cooperative awareness message (CAM) [3] is the most basic safety-related V2V message, using which, a vehicle can intimate the surrounding vehicles regarding its presence in real-time. A local database, known as the local dynamic map (LDM) [4], stores the received information and provides support for various ITS applications.

Traditional cooperative ITS largely relies on a network of on-board units (OBUs) and roadside units (RSUs). Therefore, cooperative ITS has not been able to accommodate non-connected road users, such as vulnerable road users (VRUs; e.g., pedestrians and bicycles) and legacy vehicles—neither of which have V2X devices. These non-connected users are, however, detected in the sensors equipped in the vehicle or the infrastructure environment. Cooperative perception, also known as collective or collaborative perception, integrates these non-connected users to the cooperative ITS by sharing sensor information. Cooperative perception plays a significant role in the deployment phase of V2X technologies when legacy vehicles and connected autonomous vehicles would coincide on the roads. Collective perception messages (CPMs) [5,6] is currently being developed in ETSI. The sensors can be installed either in vehicles or in the infrastructure environment, and cooperative perception works in either configuration. However, we focus on RSU-based cooperative perception in this paper because of the following reasons. Considering the above scenario wherein vehicles pass through the intersection at maximum speed, object detection coverage at the intersection must be near 100%. Thus, RSU-based cooperative perception is suitable for such a task due to the better visibility from the sensors mounted in the infrastructure environment. Moreover, the availability of the infrastructure sensors is higher than relying on the sensors mounted on vehicles, especially in the deployment phase’s low penetration ratio.

**Figure 1:**
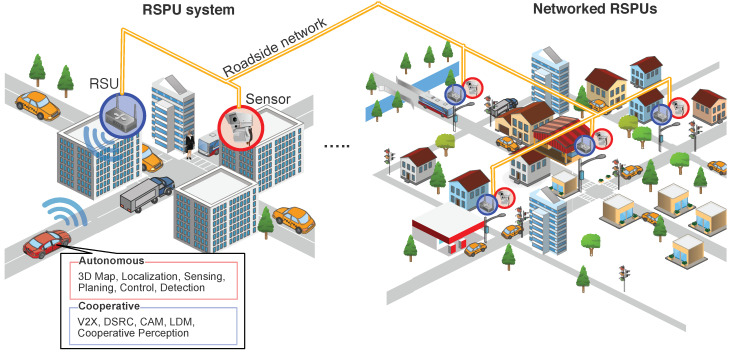
The roadside perception unit (RSPU) system and networked RSPUs.

Considering this background, we explore the two concepts introduced in Figure 1: (1) Roadside perception unit (RSPU) system and (2) networked RSPUs. On the left side of the figure, we consider a fully autonomous vehicle with cooperative ITS functions approaching an intersection. The intersection has mixed traffic with legacy vehicles and VRUs, where some of them are invisible from vehicle sensors. The RSPU system consists of sensors that detect all the objects at the intersection and RSUs that share object information via V2X communication. The right side of the figure shows networked RSPUs, which is the inter-connection of RSPUs broadly deployed at intersections.

Our study has two contributions. First, we designed and implemented an RSPU system by integrating two open-source autonomous driving and cooperative ITS software. Our software implementation is also an open-source release so as to accelerate the development of RSPUs. To our knowledge, this is the first implementation of cooperative perception that combines cooperative ITS and autonomous driving software. We also evaluated the implementation in a field test. Secondly, we developed a scheme to disseminate cooperative perception messages over broader areas by interconnecting the RSPUs through high-speed roadside networks. We also developed a priority algorithm to deliver the messages with a high packet delivery ratio (PDR) and a low delay by their respective safety contributions. The proposed scheme efficiently transfers messages in large road traffic conditions created in Tokyo and Paris’s real maps in the simulation.

The rest of the paper is organized as follows. Section 2 overviews the state of the art on cooperative perception systems for autonomous vehicles. Section 3 explores the system design and implementation of our roadside perception unit (RSPU) system for autonomous driving, called AutoC2X. In Section 4, we propose the concept of networked RSPUs, which is the inter-connection of RSPUs along a road over a wired network. Section 5 describes the evaluation of the RSPU system and the networked RSPUs through a field test, numerical analysis, and simulation. Finally, the paper is concluded in Section 6, which summarizes the study and discusses future scope of work.

## 2. State of the Art

Sensor data sharing can be classified into three categories depending on the occurrence of sensor fusion [7]: (1) In raw/low-level data sharing, raw data from the sensors is shared - such data includes point clouds from LiDAR and camera images; (2) in feature/middle-level data sharing, pre-processed data (such as a bounding box from a vision-based object detector) is shared; and (3) in object/track-level data sharing, the position information of the objects in the global coordinate system is shared.

Augmented vehicular reality (AVR) [8] systems share raw-level sensor data (point cloud) of the 3D camera depth perception sensors. Some researchers have also evaluated the realization of feature-level sensor data sharing in real vehicles [9]. In [10], the authors discussed the use of feature-level sensor data sharing to address the limited network bandwidth and stringent real-time constraints.

Object(track)-level cooperative perception has two advantages over raw- and feature-based approaches: Low wireless resource requirement and sensor agnosticism. Object(track)-level approaches such as collective perception messages (CPMs) [5,6] have received the most attention from researchers and developers because of their suitability for deployment in cooperative ITS. The bandwidth consumption in CPM was analyzed in [11,12]. A few researchers have realized object-level cooperative perception with multiple infrastructure-based sensors and transmitters [13,14]. Our previous work [15] proposed CAM-encoded infrastructure-based cooperative perception. The receivers can process the message using the same reception procedure as that of CAM. Consequently, the solution is compatible with all the products currently available in the market.

When multiple vehicles detect the same object, they all transmit the object’s information and hence introduce redundancy. A reasonable level of redundancy is useful to confirm the existence of the object. However, there is a risk of message flooding under heavy traffic. [16,17] proposed generation rules for mitigation. Some message filtering algorithms [18,19] reduce the number of messages while maintaining perception performance. [20] leveraged the trade-off between optional information and message size to save network resources. In [21], the authors introduce deep reinforcement learning that addresses the network load problem.

Security in cooperative perception is essential for deployment. In [22], the researchers attempted to examine the trustworthiness of cooperative perception by quantifying the confidence in the correctness of data by using Bayes’ theory. TruPercept [23] achieves cooperative perception through trust modeling; its dataset is openly available and includes unreliable and malicious behavior scenarios.

Researchers often evaluate the network performance of vehicular ad-hoc networks (VANETs) in the simulator. Artery [24] is a popular VANET simulation framework that combines OMNet++ (network simulator) and SUMO (traffic simulator). [25,26] extended the Artery framework for cooperative perception by adding the sensor model. However, field testing is essential to understand the real effect of sensor communication in the environment. There are only a few field tests available in the literature [27]. In [28], the authors tested V2X cooperative perception and its application to ITS by considering basic safety message (BSM) and conducting a field test involving three vehicles.

Reference [29] developed a system based on robot operating system (ROS) to visualize sensor perception and CAM and create test environments using a combination of real and virtual (simulated) objects. The cooperative automation research mobility application (CARMA) platform [30] is an open-source software that connects Autoware-based autonomous vehicles [31,32] by using US standards [33], including BSM, signal phase and timing (SPaT), and MAP. While some maneuver coordination messages are defined in CARMA, cooperative perception (i.e., sensor data sharing) is currently not supported.

In this section, we reviewed the literature on cooperative perception, including network load mitigation, security, simulation, field testing, and Cooperative autonomous driving. However, none of the studies mentioned above have developed an open-source software combining autonomous driving and cooperative ITS for RSU-based cooperative perception (i.e., RSPU). Furthermore, none of them have evaluated the scenario of networked RSPUs on a city scale.

## 3. Design and Implementation of the RSPU System

This section provides details about the design as well as the deployment of our RSPU system. More specifically, we undertook the development of AutoC2X [34] through the integration of an autonomous driving software referred to as Autoware [31,32] along with a cooperative ITS software referred to as OpenC2X [35]. Section 3.1 depicts the system design of AutoC2X through the elucidation of Autoware and OpenC2X. This is followed by Section 3.2, which outlined the execution of AutoC2X.

### 3.1. System Design of AutoC2X

**Figure 2:**
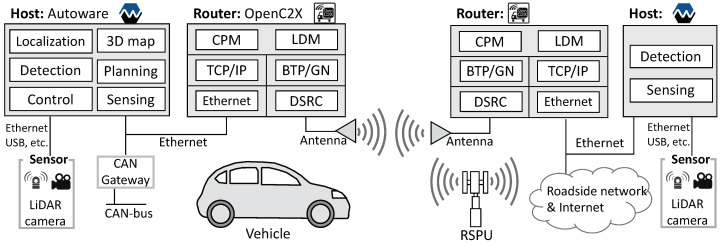
System model to integrate Autoware and OpenC2X.

Cooperative perception denotes a desirably function both at the level of the infrastructure and vehicle, which explains the rationale behind designing AutoC2X for the two nodes. The set of functionalities offered by the RSPU system is similar to that of autonomous vehicles cooperative ITS functions, Figure 2 illustrates this proposed model. We designed a system in both vehicle and RSPU, in which a host realizes perception, whereas a router takes charge of the cooperative ITS function. Put differently, OpenC2X and Autoware operate on different nodes rather than being deployed on one computer. This design was chosen since a vehicle and RSPU typically include some dedicated computers necessitating external connectivity. As a case in point, dedicated computers could be on a vehicle for navigation, mapping, and driving logs. In a similar manner, an RSPU could have separate computers serving in the capacity of traffic monitoring systems, edge servers, as well as for signals/signage. For this reason, it is a better idea to ensure that a router manages all the nodes’ external connectivity as opposed to extending all the nodes to have external connectivity, including the antenna. This configuration is also recommended by the ITS station architecture specification [1,2] also recommends such a configuration.

A key difference between RSPU and vehicle is equipment pertaining to wired connectivity and mobility. The controller area network (CAN) gateway of the vehicle helps gain control over steering, braking, and acceleration, whereas the RSPU has wired connectivity to the Internet as well as roadside networks. Moreover, the RSPU’s functionality is a subset of the autonomous vehicle, i.e., specific functionalities of autonomous driving are found to be absent. These include localization, planning, and CAN control.

#### 3.1.1. Autoware

ROS-based Autoware [31,32] is discussed in this subsection. As a middleware (open-source) framework, ROS [36] is extensively used to develop robot applications. This distributed computing platform involves topics and nodes. The nodes represent the processing module of tasks, and the nodes use topics to communicate with each other. Moreover, ROS comes up with a robust tool such as Rosbag to record and replay messages in topics. The developer can consider using Rosbag to record sensor data within the real environment before making improvements in the algorithm by using data without hardware. Furthermore, ROS is inclusive of a 3D visualization tool called RViz, that efficaciously presents the tasks’ status.

In Figure 2, it can be seen that a 3D map denotes a common digital infrastructure to operate an autonomous vehicle, particularly across urban areas. Autoware entails the use of two kinds of beforehand recorded 3D maps: Point cloud map data and vector map data. The former is used for scan matching to enable localization, whereas the latter derives the lane data. In Autoware, sensing primarily involves using 360-degree LiDAR cameras and scanners. From the LiDAR scanners, point cloud data is used for detecting surrounding objects and localization. This localization algorithm utilizes scan matching between the point cloud data sourced from LiDAR scanners and 3D point cloud maps. Generally, the normal distributions transform-based algorithm [37] is employed for the purpose of localization. Object detection is premised on point cloud data’s clustering from the LiDAR scanners, concerning with the Euclidean cluster extraction algorithm [38] is used in Autoware. Thereafter, Autoware calculates the distance between the detected objects and the own vehicle (in the following referred to as ego-vehicle). While Autoware delivers software packages for planning, decision, and prediction; however, these are currently beyond the scope of this work. Figure 2 illustrates that a CAN controller is used to perform the actuation of autonomous vehicles is with a view to manipulating steering, accelerating, and braking.

#### 3.1.2. OpenC2X

OpenC2X [35] encompasses nearly all protocol stacks in the ITS station architecture, with the exception of security entity. This system is known to support a basic service set (BSS), or offset codebook mode (OCB) mode, also taking into consideration decentralized control of congestion to comply with the protocol behavior based on alterations in vehicle density. In the transport and network layer, the system lends partial support to GeoNetworking (GN) as well as basic transport protocol (BTP). Despite the fact that it does not tackle forwarding, the GN and BTP headers get added to the sending packets. CAM, LDM, and the decentralized environmental notification message (DENM) are incorporated in the facilities layer. It is notable that CAMs are sporadically triggered from 100 to 1000 ms, in accordance with the standards. Moreover, a user is capable of using connected ITS applications, e.g., collision avoidance applications, or triggering DENMs from the web interfaces. The entire content received from CAMs and DENMs is stored in LDM stores. Additionally, OpenC2X offers a web-driven graphical user interface for visualizing the status of the ITS station.

### 3.2. Implementation of AutoC2X

**Figure 3:**
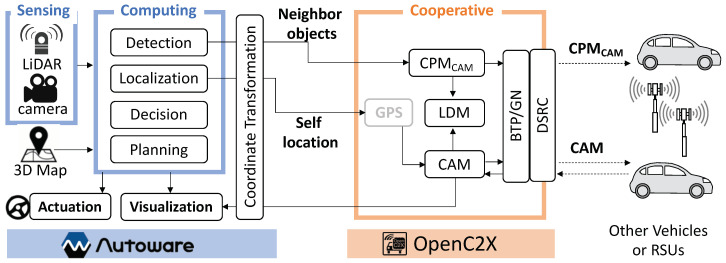
AutoC2X overview.

Figure 3 provides an overview of AutoC2X’s architecture. C++ language was used to develop AutoC2X through the extension of OpenC2X (standalone v. 1.5) and Autoware (v. 1.11.1). The source code can be accessed at https://github.com/esakilab/AutoC2X-AW.

All functions of AutoC2X are explained here. Certain features such as planning, decision, and localization are not available in the RSPU setting. In the figure, these boxes denote the functions. Arrows from right to left (→) and left to right (←) signify the receiver side sequence and sender-side, respectively. After initiating a system, Autoware reads the 3D maps and begins receiving the sensor data. The system localizes the ego-vehicle and then gets the neighboring objects detected. Coordinates of the ego-vehicle, as well as detected objects, then get transformed from local to the global coordinate system before being sent across to the OpenC2X router using TCP/IP over Ethernet. OpenC2X gets the ego-vehicle’s coordinates encoded in CAM and the coordinates of neighboring objects in the CAM-encoded collective perception message ($CPM_{CAM}$) form, before transmitting them over DSRC with the BTP/GN header. Simultaneously, the LDM stores the data.

Upon receiving CAMs and $CPM_{CAM}$s, the OpenC2X router extracts the information relating to the neighboring objects from the messages before storing it in the LDM. OpenC2X then transmits the objects’ coordinates to the Autoware. Finally, the object-related data extracted from the V2X communication gets visualized in RViz following the coordinate transformation. Data with regard to planning and decision function is not fed back to Autoware. The realization of this feedback forms an important component of our future work.

#### 3.2.1. Localization

Autoware and OpenC2X employ scan matching and GPS for localization. Put differently, the function of localization is duplicated in the host and subsequently the router in the ITS station. The localization function of Autoware is employed throughout the ITS station in the planned system due to the high localization accuracy of scan matching, which is generally less than 10 cm. Furthermore, in comparison to GPS, scan matching is found to be more robust in the urban environment. Thus, in Autoware, the ego-vehicle location is published in the topic of /ndt_pose in the developed system, before being transmitted to OpenC2X in the aftermath of coordinate transformation. Rather than the GPS coordinates, OpenC2X makes use of the received position.

#### 3.2.2. Cooperative Perception

Autoware uses a LiDAR and a camera to detect the surrounding objects. Following sensor fusion, the detected objects’ information gets published under the domain of /detection/objects. In addition to performing the coordinate transformation, the planned Autoware extension also dispatched information to OpenC2X. Subsequently, the OpenC2X extension adds the object information (detected) to a queue before encoding the most recent information within the queue into a $CPM_{CAM}$ at a frequency of 10 Hz. Currently, information available in the extension comprises the following: Latitude, timestamp, speed, longitude. On the other hand, ITS station ID, as well as other fields of CAM, are left “unknown.” In the future, if additional sensors are used for estimating additional information, such as heading and vehicle length, it is possible to populate more fields. Random numbers are then assigned to the ITS station IDs of the traced objects.

#### 3.2.3. Coordinate Transformation

Autoware utilizes the self-centered local coordinate system to maintain the coordinates while OpenC2X makes use of the geodetic reference system involving longitude and latitude. This is what makes it necessary to perform a coordination transformation. The planned Autoware extension initially changes the local coordinate system in the Universal Transverse Mercator, or UTM, coordinate system in accordance with the 3D map. The detected object’s location in the UTM coordinate system (XobjYobj) is expressed as

(1) $$\begin{array}{cc} \left(\begin{array}{c} X_{obj}\\ Y_{obj} \end{array}\right) & =\left(\begin{array}{c} X_{ego}\\ Y_{ego} \end{array}\right)+R\left(\alpha \right)\left(\begin{array}{c} x_{obj}\\ y_{obj} \end{array}\right)\\ & =\left(\begin{array}{c} X_{ego}\\ Y_{ego} \end{array}\right)+\left(\begin{array}{cc} \cos\alpha & -\sin\alpha \\ \sin\alpha & \cos\alpha \end{array}\right)\left(\begin{array}{c} x_{obj}\\ y_{obj} \end{array}\right)\\ & =\left(\begin{array}{c} X_{ego}+x_{obj}\cos\alpha -y_{obj}\sin\alpha \\ Y_{ego}+x_{obj}\sin\alpha +y_{obj}\cos\alpha \end{array}\right), \end{array}$$

where (XegoYego) denotes the ego-vehicle’s location in the UTM coordinate mechanism published in /ndt_pose; on the other hand, R(α) denotes the ego-vehicle’s rotation concerning the UTM coordinate system, published in /tf; and (xobjyobj) signifies the detected objects’ relative portion from the ego-vehicle its heading is in the X-axis, published in the topic of /detection/objects. Thereafter, the extension tuns the UTM coordinate system into the geodetic reference system 1980 (GSR80) by using PROJ library [39].

In contrast, when the extension gets a $CPM_{CAM}$ within the GSR80 form from the OpenC2X, the extension turns the coordinates into the local coordinate system for getting it handled in the Autoware.

#### 3.2.4. Visualization

**Figure 4:**
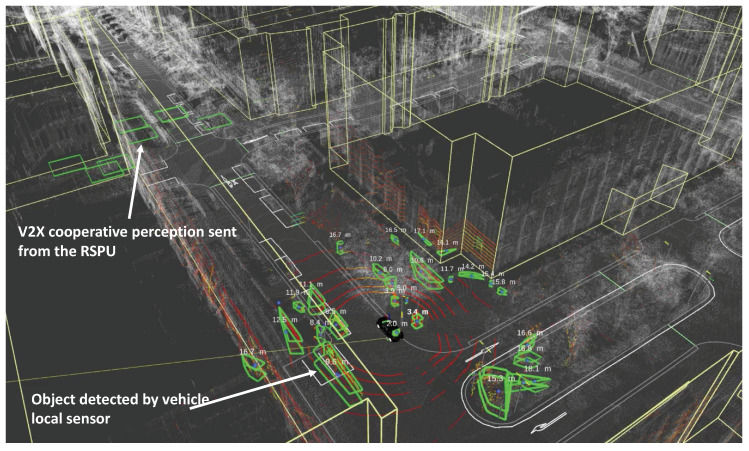
Screenshot of RViz with AutoC2X.

The Autoware extension gets the detected object information published from the received $CPM_{CAM}$ to /detection/objects. RViz visualizes the objects in the topic, as shown in Figure 4. In the figure, it is evident that an autonomous vehicle approaches an intersection. Around the vehicle, the green boxes highlight the objects that the local LiDAR sensor detect, whereas the green squares in close proximity to the intersection illustrate the objects obtained from the V2X cooperative perception. Currently, we utilize a square wherein the latitude and longitude of the object are at the center with a view to facilitating visualization. Depending on the vehicle type and size, it is possible also to visualize these objects using myriad shapes, in case the received $CPM_{CAM}$ (or CAM) possesses this information.

## 4. Networked RSPUs

The RSPU system mentioned in the previous section entails a limited dissemination area to disseminate cooperative perception messages. For this reason, we designed a networked RSPUs by linking the RSPUs via a roadside network (high-speed) to widen the message dissemination range (We called this scheme as Grid Proxy CAM in our previous work [40]).

The following requirements were identified to design this solution:

- Real-time message delivery: Real-time message transmission makes it possible to track dynamic information about vehicles, including acceleration, velocity, and position. For example, standard CAMs are known to transmit at 1 ∼ 10 times/second. Thus, a solution must be able to frequently transmit dynamic information of the vehicle and minimize deferments in sensing/transmitting messages. It is necessary to design solutions to directly deliver messages on edge with a view to bypassing cloud systems as well as the internet.
- Prioritizing messages: It is necessary for the solution to send messages on a frequent basis on a broader path. As road traffic goes up, the total number of delivered must also go up. Ideally, the wired network on the roadside should possess an adequate capacity for several academic messages at peak conditions of road traffic. That being said, the wireless link is likely to hit a point of saturation in cases excessive messages reach it. Hence, it is necessary to get messages prioritized with regard to significance on the basis of their contributions to safety.

### 4.1. System Design of Networked RSPUs

**Figure 5:**
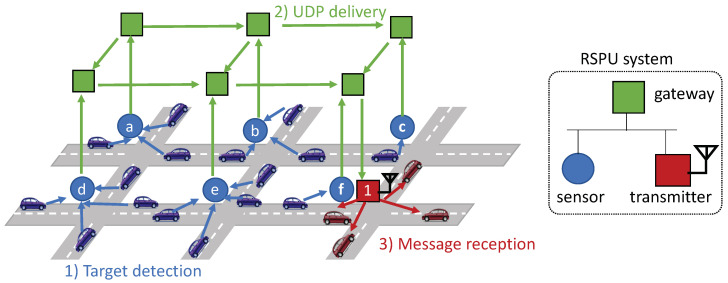
Overview of the networked RSPUs.

Figure 5 provides an overview of the RSPUs, each of which includes a gateway, transmitter as well as sensor. In each of these intersections, an RSPU is deployed. Figure 5 illustrates red vehicles receiving blue target vehicles’ $CPM_{CAM}$ through transmitter 1 from all sensors in the adjoining areas (a−f).

Figure 5(1) shows that after detecting a pedestrian or target vehicle, the sensor is capable of generating $CPM_{CAM}$ through the information sourced from the object. The RSPU utilizes the transmitter for broadcasting the message. Figure 5(2) illustrates that simultaneously, the user datagram protocol, or UDP, is used to send $CPM_{CAM}$ to adjacent RSPUs in the range that the operator of the road infrastructure has already configured. It is assumed that all RSPUs are provided with a list comprising neighboring RSPUs’ IP addresses beforehand. UDP packets are transmitted at the peak frequency of 10 Hz -the as per the specification of CAM. Figure 5(3) shows that BTP and GN headers are added by the transmitter after the UDP packet is received. Subsequently, the transmitter places the $CPM_{CAM}$ on the queue of the IEEE802.11p interface, whose MAC layer employs the mechanism of the IEEE 802.11e-based enhanced distributed channel access or EDCA [41]. In this manner, the classification of $CPM_{CAM}$ in the AC_BE class also occurs. The next section elucidates the policy of queuing priority.

### 4.2. Distance Priority Algorithm

Under a broader cooperation perception, the more efficient and safer ITS application does get supported, albeit with an increase in the number of messages. In addition to lowering PDR, it also exacerbates the element of delay. To illustrate, it is not possible for a transmitted to broadcast all messages amidst heavy traffic due to the saturation of its wire link’s capacity; 3∼27 Mbps is the data rate associated with IEEE802.11p. If the $CPM_{CAM}$s exceeds the wireless link’s sending rate of messages sent to the transmitter, the queue received further messages, thus resulting in increased delay and waiting time and rendering the received information redundant.

To overcome these challenges, our approach entails the prioritization of messages on the basis of their contribution to safety. Based on the volume of road traffic, there is variation in the total number of objects that are detected (number of messages). In case there is adequate capacity for broadcasting more $CPM_{CAM}$s, the ones with more information on the distant object join the queue.

$d_{max}$, is the peak distance of the transmitter, whereas the $r_{qo}$ denotes the constantly monitored ratio of queue occupancy. After $CPM_{CAM}$, is received, the distance *d* is calculated between the transmitter position and that of the detected object within the aforementioned message. This is done with a view to ascertaining whether the packet must join the queue or dropped in the following manner:

(2) $$r_{qo}<1-\frac{d}{d_{max}}.$$

If ‘’true” is the value returned by the equation, the queue witnesses the addition of $CPM_{CAM}$;. Accordingly, $CPM_{CAM}$s containing additional information on distant objects gets dropped when the value of $r_{qo}$ is adequately large; its value is also reduced by the algorithm to reduce end-to-end deferments. Notably, the transmitter begins dropping $CPM_{CAM}$s with additional information (distance related) prior to the queue getting full. However, it is able to maintain sufficient space for information related to future usage.

## 5. Evaluation

Subsequently, we undertook the evaluation of the AutoC2X-based RSPU system using a vehicle and hardware. We followed it up with a numerical analysis of the networked RSPUs before carrying out simulation experiments. Section 5.1 chronicles the AutoC2X-based RSPU system’s experimental evaluation using a field testbed and an indoor testbed. Section 5.2 elucidates the networked RSPUs’ numerical analysis with a view to estimating network performance in large-scale deployments. Section 5.3, explains our analysis of networked RSPUs with several scenarios using a network simulator.

### 5.1. Experimental Evaluation

**Figure 6:**
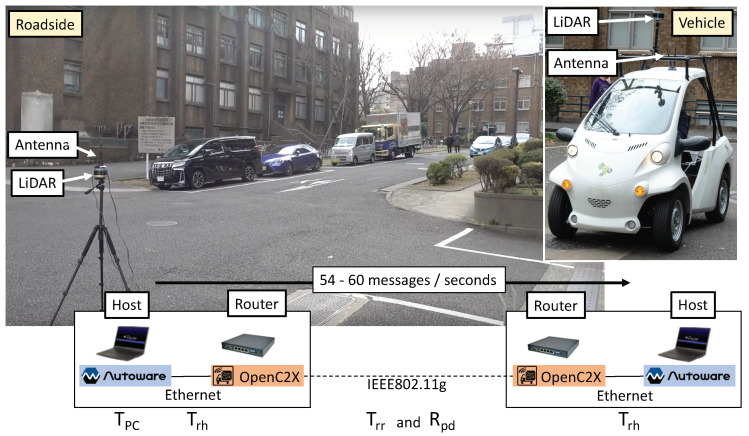
Experimental setup.

As shown in Figure 6, we took into consideration a scenario wherein an RSPU stated at an intersection gets the $CPM_{CAM}$s broadcasted to the surrounding vehicle (at Hongo campus in the University of Tokyo, Japan). The same equipment (an OpenC2X router, an Autoware host, an antenna, and a LiDAR sensor) was involved in both the RSPU and the vehicle. With an Intel Wi-Fi 6 AX200 module, the routers were APU4C4-embedded routers wherein Ubuntu 18.04.3 LTS is deployed. In addition, the hosts included laptop PCs (CPU Core i7 8-cores) with 32 GB (vehicle host) RAM memory and Ubuntu 16.04.5 LTS and 16 GB (RSPU host). The vehicle and the RSPU were equipped with Velodyne VLP-16 LiDAR sensors. As per the Japanese regulations, the tests involved the use of the IEEE 802.11g ad-hoc mode.

Figure 6 shows that the total delay *T* and PDR $R_{pd}$ were viewed as the metrics of evaluation. $R_{pd}$ denoted the PDR between the routers since there was no packet loss in the ITS station (i.e., Ethernet link) across all experiments. $R_{pd}$ was measured by comparing the LDM entries of the sender and receiver after the experiments. The total delay *T* is expressed as

(3) $$T=T_{pc}+T_{hr}+T_{rr}+T_{rh},$$

where $T_{pc}$ signifies the delay in Autoware. On the other hand, $T_{hr}$, $T_{rr}$, and $T_{rh}$ denote the transmission delay from the host to the router, between routers, and from the router to the host, respectively. $T_{pc}$ is inclusive of the processing delay in Autoware relating to the clustering of detection, point cloud, and the objects’ localization; the calculation of this value is made by taking into consideration the gap between the point cloud’s published time and the time of the router’s message. $T_{hr}$, $T_{rr}$, and $T_{rh}$ are calculated by taking into consideration the round-trip time for each link. Upon the message’s message, the proposed extensions for both Autoware and OpenC2X return the same message to the sender for measuring the round-trip time.

#### 5.1.1. Indoor Test

As shown in Figure 6, we conducted the indoor test by placing the routers and hosts on a desk. Between the routers, the distance was nearly 3 m. For the RSPU, the Rosbag was recorded at an intersection at the Hongo campus. For the vehicle, it was recorded at the school. The duration of Rosbags was 4 min 35 s, which were replayed in the vehicle hosts and RSPU. Meanwhile, an average of 57 messages was created the RSPU as per second. All measurements were made as many as five times.

**Figure 7:**
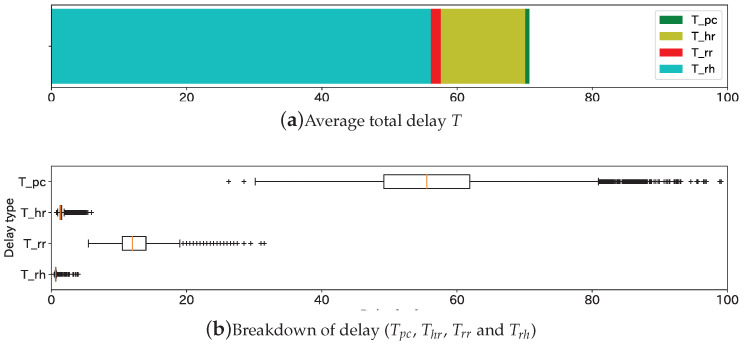
Total delay and the breakdown. (**a**) Average total delay *T*, (**b**) Breakdown of delay ($T_{pc}$, $T_{hr}$, $T_{rr}$ and $T_{rh}$).

Figure 7 illustrates the findings of the indoor experiment. Figure 7a shows that the overall average was T was 70.7 ms, and Tpc occupied 80% of it. As illustrated in Figure 7b, the peak delay relating to $T_{pc}$, $T_{hr}$, $T_{rr}$, and $T_{rh}$ were nearly 80 ms, 3 ms, 20 ms, and 3 ms, respectively. Therefore, the autonomous vehicle was able to receive the cooperative perception message in nearly 100 ms even in the worst-case scenarios; 10 Hz, which is also the maximum frequency for CAM, is the frequency of point cloud measurement. For this reason, the result demonstrates that the proposed system is capable of delivering the message both within the point cloud measurement interval and within the CAM interval.

#### 5.1.2. Field Experiment

**Figure 8:**
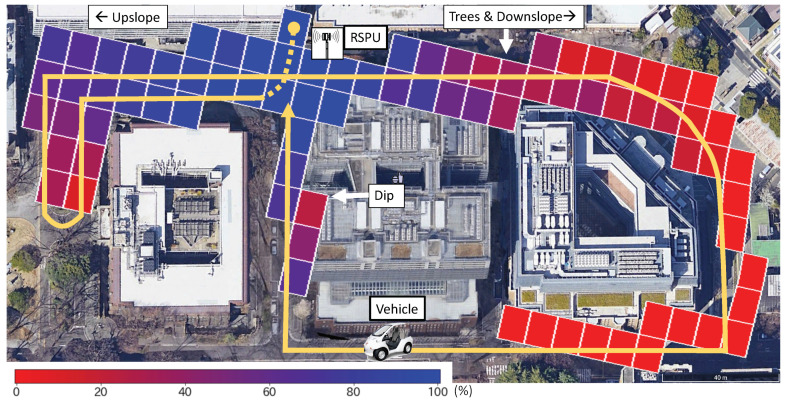
Packet delivery ratio $R_{pd}$ in field experiment.

Figure 8 illustrates the location of the RSPU as well as the driving route of the vehicle. In the figure, the vehicle drove along the yellow line in at nearly 3 km/h several times in a total time of 1000 s. The figure also depicts the experimental results. The tiles’ color is indicative of the value of $R_{pd}$ at the specific location. The absence of titles suggests that the location did not receive any message.

In the majority of cases, $R_{pd}$ gradually decreased as the distance increased. Additionally, the buildings attenuate the transmission of messages, thus causing a radical decline in the PDR value. The left and right sides of the RSPU correspond to an upslope and a downslope with several trees. Consequently, the left side has a better LOS, leading to a better PDR. The result demonstrates that the value of PDR is over 80% when the vehicle is at 30 m and also has LOS to the RSPU. Finally, the value of PDR is observed to degrade at the bottom of the dip located in the figure’s central part.

### 5.2. Numerical Analysis

A mathematical evaluation was carried out about the RSPUs’ underlying performance with regard to PDR and delay in delivering the messages. To begin with, we undertook a calculation of the throughput of $CPM_{CAM}$ across IEEE802.11p. In these calculations, Table 1 provides a summary of values, symbols, and variables. The following equation denotes the average interval necessary for sending a $CPM_{CAM}$ packet through $t_{cpm}$, a wireless channel:

(4) $$t_{cpm}=t_{DIFS}+b+t_{send},$$

where $t_{DIFS}$ denotes the interval of distributed interframe space, *b* signifies the backoff time, whereas $t_{send}$ denotes the transmission’s duration.

$t_{DIFS}$, the interval of DIFS, denotes the time for waiting after the channel becomes an ideal state. It is shown by the following equation:

(5) $$t_{DIFS}=AIFSN\times slen_{11p}+t_{SIFS}.$$

As far as the AC_BE class is concerned within the EDCA, six is the arbitration interframe space number (AIFSN) upon the activation of OCB [42]; *b*, the backoff time denotes the random waiting time that the following equation denotes

(6) $$b=CW_{ave}\times slen_{11p},$$

where the value of CW, the contention window size, is 0 ∼ 15 [42]. The average contention windows size ($CV_{ave}$) = 7.5; for this reason, b= 0.0975 ms.

A $CPM_{CAM}$’s duration of transmission is denoted by the following equation:

(7) $$\begin{array}{cc} t_{send} & =t_{switch}+t_{pre}+t_{sig}\\ \; & \;+t_{sym}\times ceil\left(\frac{16+P_{cpm}+6}{N_{DBPS}}\right), \end{array}$$

where $t_{switch}$ denotes the switch time between Rx and Tx, On the other hand, $t_{pre}$ refers to the duration of physical layer convergence protocol, or PLCP, $t_{sig}$ signifies the PLCP signal’s total duration, $N_{DBPS}$ denotes the data bits per symbol of orthogonal frequency-division multiplexing (OFDM), $t_{sym}$ represents the interval of symbol, whereas ceil() refers to a function that returns the smallest sized integer equal to or higher than a particular number. In case 18 Mbps is the data rate, the utilization of 16QAM is specified for the modulation scheme following which the code rate is 3/4. Thus, from Equation 7, $t_{sym}=$ = 8 μs and $N_{DBPS}=$ 144 bits, which implies that $t_{send}=$ 0.081 ms

Based on Equation 4, the interval of time (average) required for successful transmission of CPM packet is denoted by $t_{cpm}=$ 0.2885 ms. On average, a $CPM_{CAM}$ gets sent across 0.2885 ms, thus implying in an efficacious throughput of CPM on of approximately 2.73 Mbps.

Accordingly, it is possible to get the data rate calculated at which the transmitter is capable of broadcasting CPMs without keeping any within the queue. In case all RSPUs, on average, trace ten vehicles, it is also capable of broadcasting messages from nearly 40 adjacent RSPUs. This means that queuing would not commence until RSPU detects over ten vehicles and when messages are being sent by over 40 neighboring RSPUs. This will lead to a situation where end-to-end PDR would reduce, whereas the corresponding delays will increase in scenarios where the distance priority-based algorithm is not implemented.

### 5.3. Simulation Analysis

To evaluate communication performance about PDR and delay under several situations, the RSPUs were implemented over the framework titled Artery (https://github.com/riebl/artery) which denotes an extension of vehicular network simulation framework (Veins http://veins.car2x.org) wherein the network is simulated on OMNeT++ (https://omnetpp.org) with the simulation of vehicular traffic taking place on SUMO (http://sumo.dlr.de/).

**Figure 9:**
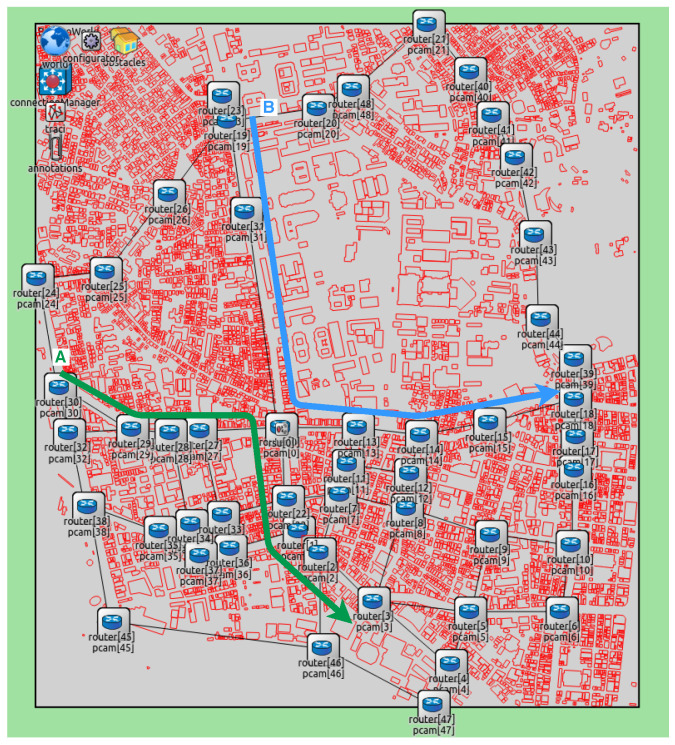
Map of Tokyo.

**Figure 10:**
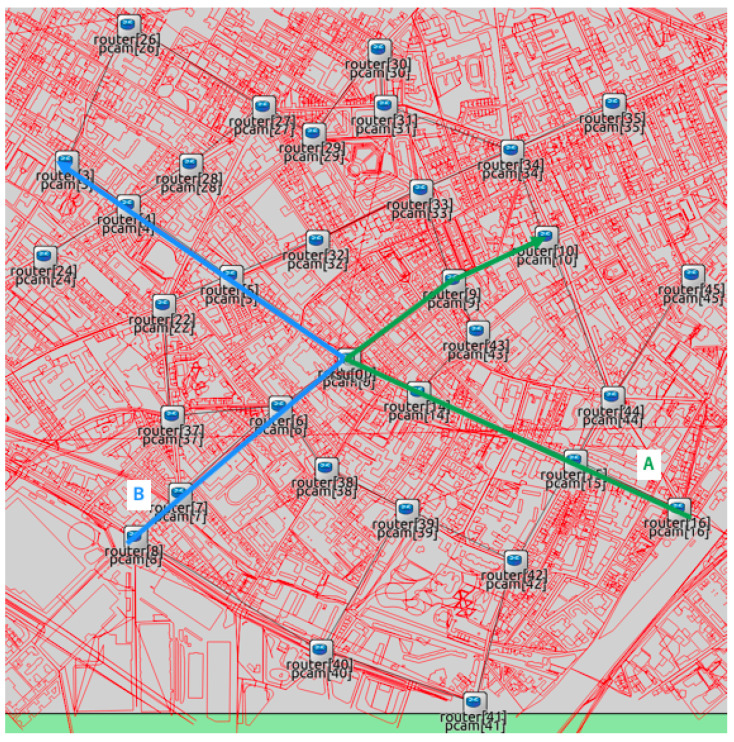
Map of Paris.

We conducted the simulation experiments by utilizing maps covering the University of Tokyo and the region near Paris, as shown in Figure 9 and Figure 10. OpenStreetMap wiki was used to take both maps. All chosen intersections had an RSPU. In addition, all RSPUs were interconnected through an Ethernet cable. The RSPUs of Paris and Tokyo maps were 32 and 49, respectively, with each maintaining the routes to others via routing information protocol. The red lines denote obstacles or buildings. In addition to attenuating wireless radio, these obstacles can also be seen to be blocking the line of sight between the source and destination codes. All building edge impedes a path that the signal attenuates by 9 dB; 0.4 dB per meter attenuates the signal that passes via a building. The results of experiments encompassing both scenarios were as follows: (1) Isolated use of network RSPUs, and (2) combination of distance priority algorithm and using networked RSPUs. Table 2 lists the simulations’ parameters.

The radio frequency, data rate, reception sensitivity, and transmission power were 5.89 GHz, 18 Mbps, −89 dBm, and 126 mW, respectively. The peak range of radio was restricted to 150 m for reflecting the usual limitations of radio coverage in urban areas. The frequency of all messages generated by RSPUs was 10 Hz. The length of the queue was 1000 packets, while the peak distance was 1000 m. The speed of simulated vehicles traveling via intersections on two routes was 50 km/has shown in Figure 9 and Figure 10. There were no stoppages as each vehicle traveled since the roads were not assumed to have any traffic signals; every second, a couple of vehicles passed via the intersections. This vehicle traffic’s frequency is the traffic that finds mention in the Japanese Police Department’s official statistics of traffic outlined in the corresponding map shown in Figure 9.

After detecting 5, 10, or 15 vehicles at the end of every 0.1 s, each RSPU transmitted UDPs across to all adjoining RSPUs. Under the assumption that the coverage of sensor detection was 50 m, the sensors encompassed 200 m in four directions that pointed outward from the intersection. Thus, the traffic rate in the scenarios was fixed at 25, 50, and 75 vehicles/km for the purpose of reflecting the tracing of 5, 10, and 15 vehicles per 200 m, respectively.

The performance of communication was measured at intersections, wherein both routes crossed. To take the measurements, an evaluation node deployed in close proximity to the intersection was deployed for receiving $CPM_{CAM}$s from all RSPUs via IEEE802.11p. Both the delay and PDR were evaluated from the source RSPU to the assessment node. All Each simulation was carried out 100 times each at random speeds to calculate the standard deviations and averages for the findings. The duration of each simulation was 15 s. Findings from 5–10 s were used to ensure the measurement of communication performance was made in a steady condition.

#### 5.3.1. PDR Evaluation

**Figure 11:**
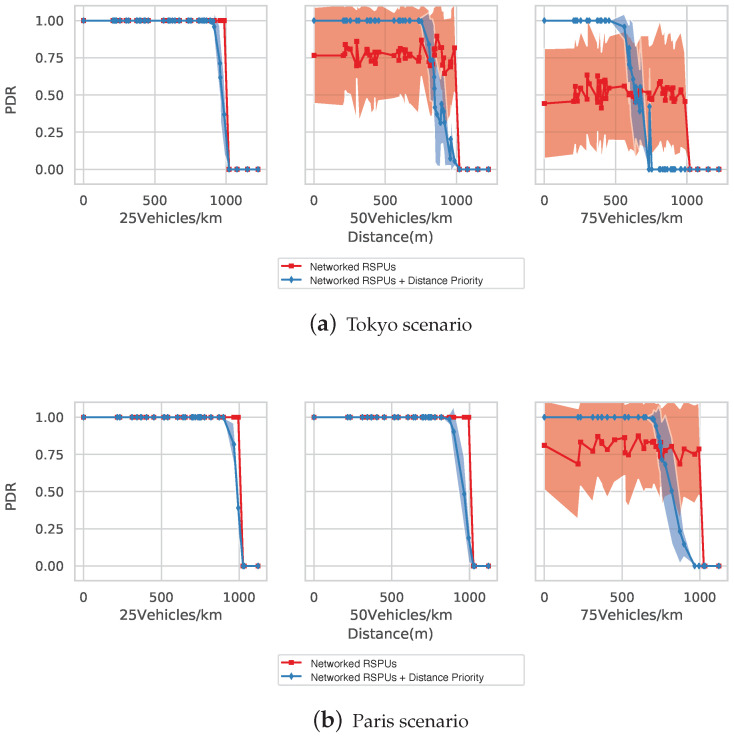
Evaluation of packet delivery ratio (PDR). (**a**) Tokyo scenarios, (**b**) Paris scenario.

Figure 11a illustrates the simulation of Tokyo Map’s PDR with densities being 25, 50, and 75 vehicles/km. Blue and red lines denote the RSPUs scenarios with and without the distance priority algorithm, respectively, also reflective of the average PDRs. The colored zones below/above show the standard deviations. In line with expectations, the two RSPUs schemes were able to transmit messages across greater distances as compared to the lone RSPU scenario wherein the packet was not delivered further than the range of wireless under any scenarios of vehicle density.

The RSPUs scenario was able to maintain a PDR of 100% across all distances when the vehicle’s density was 25 vehicles/km. On the contrary, those with distance priority algorithm scenario were able to replicate the maintenance of PDR till a distance of 900 m, beyond which a gradual decline to 1000 m was observed since the algorithm disregarded information provided by distant transmitters for maintaining space for further information from closer proximity.

The RSPUs scenario was able to maintain a PDR of 70% when the vehicle’s density was 50 vehicles/k. However, big standard deviations were seen for these reasons. The packets that arrived initially at the stated RSPU did reach the receiving node. However, the subsequent ones risked a decline in packets in accordance with the ratio of queue occupancy. With the simulation reflecting the randomness of vehicle detection (in terms of timing), the packet-drop targeted packets from more than one distance, thus increasing the standard deviation. The distance priority algorithm’s application led to a 100% PDR, with the distance being 740 m. Subsequently, a gradual decline in PDR was observed until a distance of 990 m; the detection timing’s randomness also led to a comparatively big standard deviation.

Nearly 50% of the messages got lost across the distances when the vehicle’s density was 75 vehicles/km. However, the aforementioned algorithm’s application ensured that all messages were delivered across a 450 m range. Thereafter, there was a gradual decline of PDR up to 800 m.

The Paris map’s PDR is shown in Figure 11b. When vehicle densities were 25 and 50 vehicles/km, there was 100% PDR under the non-priority situation across all covered distances due to fewer RSPUs being deployed in the intersections. The algorithm drops messages from far away transmitters (850 and 900 m) for ensuring the information’s reservation in closer transmitters. When the density was 75 vehicles/km, a 100% PDR was maintained by this distance priority algorithm within a range of 650 m, with the non-priority situation being unstable across all distances.

As indicated by the above findings, the networked RSPUs system not only broadens the range of message transmission but also allow the algorithm to maintain a message delivery rate of 100% for information obtained from RSPUs.

#### 5.3.2. Delay Evaluation

In the Tokyo simulation, delays can be seen when the vehicle densities are at 25, 50, and 75 vehicles/km. The delay was nearly 10 ms under the two RSPUs scenarios till the peak distance of 1000 m. However, a delay of 290 ms was seen in the no-priority scenario calculated as per Section 5.2. The delay was reduced by the algorithm to 40 ms in a range of 740 m, wherein the PDR was observed at 100%. Subsequently, the delay rose to 990 m from 740 m as packet drop took place in this range. The linked RSPUs scenario witnessed a delay of 290 ms at densities of 75 vehicles/km. The delay was reduced by the algorithm to 100 ms within 450 m, wherein the PDR was observed at 100%. Therefore, the delay rose to 800 m from 450 m.

**Figure 12:**
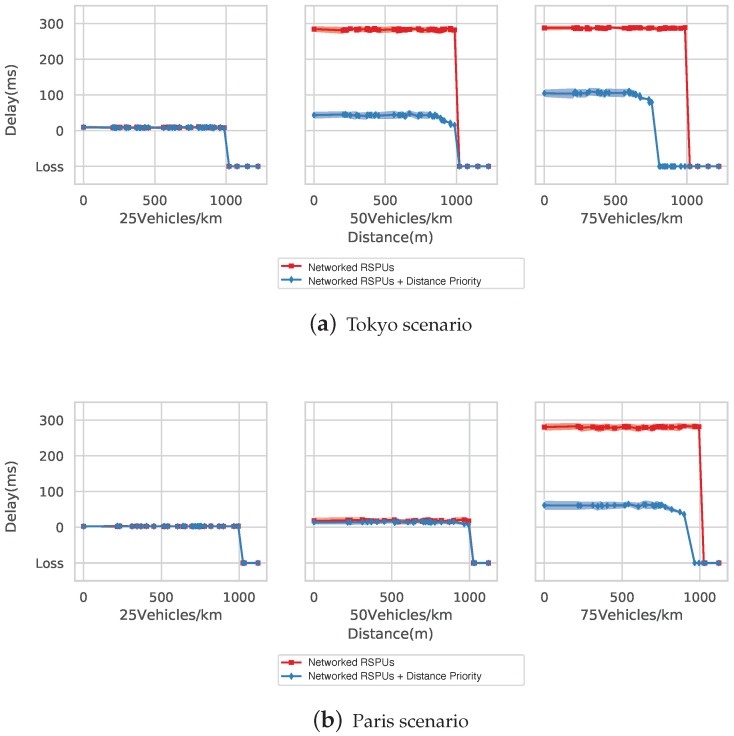
Evaluation of delays. (**a**) Tokyo scenarios, (**b**) Paris scenario.

Figure 12b illustrates the delays in simulations of the Paris map. A delay of nearly 10 ms was seen in both situations at 25 vehicles/km. However, this delay rose to nearly under both situations at densities of 50 vehicles/km. The delay was approximately 290 ms at 75 vehicles/km in the absence of the planned algorithm, with the distance priority-based algorithm decreasing the deferment to 65 ms.

According to the above findings, the linked RSPUs system is capable of extensively delivering the messages. In addition, it can also be seen that the distance-based priority algorithm helps decrease the delay for information obtained from closely situated RSPUs.

## 6. Conclusion and Future Work

In this paper, we discussed AutoC2X, an open-source software we developed to enable both vehicle-based infrastructure-based cooperative perception. We also described the field experiments conducted on the AutoC2X-based RSPU system proposed in the study. Experimental results showed that, even in the worst case, the delay in receipt of cooperative perception message was only 100 ms with Wi-Fi. The proposed system is independent of the access layer technologies, and we believe that the latency will be better using C-V2X or 5G. To realize broader cooperative perception, we proposed and discussed the concept of networked RSPUs, which is the inter-connection of RSPUs along a road over a wired network. To balance the trade-off between wider message dissemination, delivery delay, and PDR, we proposed an algorithm that provides higher priority to cooperative perception messages corresponding to areas closer to the receivers. The evaluation results demonstrated that our proposed scheme successfully widen the perception range and delivers messages with a high delivery ratio and low latency, especially under massive road traffic.

In the future, we plan to work on the following: First, further development of AutoC2X is necessary to feedback the V2X information to autonomous driving. For example, prediction and planning must take advantage of cooperative perception. Second, the proposed networked RSPUs can benefit from a CPM generation rule [11] that mitigates latency and packet loss. Further research is necessary to verify the applicability of the technique to networked RSPUs. Third, scenarios with multiple sensors and multiple transmitters require further investigation with regard to both the RSPU system and networked RSPUs.

## Figures and Tables

**Table 1:** MAC and Physical Layers’ Parameters.

Layer	Variable Type	Symbol	Value
MAC	AIFSN with OCB	AIFSN	6
	Slot length of IEEE802.11p	$slen_{11p}$	0.013 ms
	SIFS interval	$t_{SIFS}$	0.032 ms
	Contention windows size	CW	0∼15
	Average of CW	$CW_{ave}$	7.5
PHY	Switch time between Tx and Rx	$t_{switch}$	0.001 ms
	PLCP preamble duration	$t_{pre}$	0.032 ms
	Duration of PLCP Signal	$t_{sig}$	0.008 ms
	Symbol interval	$t_{sym}$	8 μs
	Number of data bits per symbol	$N_{DBPS}$	144 bits
Other	Payload Length of $CPM_{CAM}$	$P_{cpm}$	680 bits

**Table 2:** Parameters of Simulations.

Type	Variable Name	Value
Radio	IEEE802.11p datarate	18 Mbps
	Attenuation per building edge	9 db
	Attenuation through building	0.4 db/m
	Radio range	150m
RSPU	Queue length	1000 packets
	Maximum distance ($d_{max}$)	1000 m
	$CPM_{CAM}$ frequency	10 Hz
Vehicle traffic	Vehicle speed	50 km/h
	Vehicle num per sec per intersection	2
	CAM frequency	10 Hz

## Abbreviations

The following abbreviations are used in this manuscript:

BTP	Basic Transport Protocol
CAM	Cooperative Awareness Message
CAN	Controller area network
CPM	Collective Perception Message
$CPM_{CAM}$	CAM-encorded collective perception message
DSRC	Dedicated short range communication
ETSI	European Telecommunications Standards Institute
GN	GeoNetworking
GNSS	Global navigation system satellite
LDM	Local dynamic map
PDR	Packet delivery Radio
RSPU	Roadside perception unit
RSU	Roadside unit
UTM	Universal Transverse Mercator
V2X	Vehicle-to-everything
VANET	Vehicular Ad-Hoc Network
